# Reimagining Caregiving Roles and Challenging Gender Norms: A Husband’s Experience with Caring for Wife Living with Dementia

**DOI:** 10.1177/1097184X251351628

**Published:** 2025-06-12

**Authors:** Shreemouna Gurung, Katherine Bertoni, Lucy Kervin, Kishore Seetharaman, Koushambhi Basu Khan, Jennifer Baumbusch

**Affiliations:** 1 Department of Gerontology, 1763 Simon Fraser University, Vancouver, BC, Canada; 2 School of Nursing, 8205 University of Victoria, Victoria, BC, Canada; 3 School of Nursing, 8166 University of British Colombia, Vancouver, BC, Canada

**Keywords:** spousal caregiving, men caregivers, dementia care, community-dwelling, longitudinal qualitative study

## Abstract

Spousal care is a prominent support for persons living with dementia, yet the experiences of men who provide spousal care are understudied. This research explored the experiences of husbands caring for wives with progressive dementia in a community setting. In this paper, the experience of a 63-year-old retired man caring for his wife diagnosed with early onset dementia was explored using a longitudinal qualitative case study approach. Reflexive thematic analysis of interview and narrative diary data produced two themes: (1) The Practical Realities of Caregiving, underscoring the everyday challenges of caregiving and (2) The Emotional Landscape of Caregiving, highlighting the complex emotional journey of caregivers. Findings emphasize the emotional challenges faced by men caregivers and highlight the need for supports that are tailored to men’s unique experiences and emotional needs.

## Introduction

As the population ages, the global prevalence of dementia is expected to increase to 82 million by 2030 and 152 million by 2050 (Alzheimer’s Disease International 2015). In Canada, dementia prevalence is expected to reach almost one million by 2030 and over 1.7 million by 2050 (Alzheimer Society of Canada 2022). In 2020, nearly 600,000 Canadians were living with dementia and, in 2022, over 350 Canadians were diagnosed with dementia daily (Alzheimer Society of Canada 2022). Dementia is an umbrella term describing multiple diseases. It encompasses a spectrum of symptoms resulting from chronic and progressive changes in the brain, including memory loss, alteration in mood or behaviour, difficulties with cognition (e.g., thinking, speaking, problem-solving, and recognition), and physical manifestations (e.g., changes in gait, posture, muscle strength, and coordination) (Alzheimer Society of Canada 2024; Centers for Disease Control and Prevention 2023).

Women are disproportionately affected: nearly two-thirds of older adults living with dementia in Canada and the United States are women, with numbers in Canada projected to reach one million by 2050 (Alzheimer Society of Canada 2022; Alzheimer’s Association 2024). Globally, the ratio of dementia in women compared to men is projected to be 2:1 (GBD 2019 Dementia Forecasting Collaborators 2022). Most informal care for older women living with dementia is provided by their spouses/partners or adult children (Canadian Institute for Health Information [CIHI] 2018). While not all women with dementia are cared for by men, the disproportionate prevalence of dementia among women suggests a likely increase in men caregivers. In the context of heterosexual partnerships, this trend is especially relevant for men who provide spousal care, given common caregiving patterns and gendered expectations (Robinson et al. 2014; Schaffler-Schaden et al. 2021).

Informal caregivers—typically unpaid family or friends—provide an average of 26 hours of care per week, largely within the home (CIHI 2018). As a person’s dementia progresses, their care demands intensify, contributing to caregiver burden, including financial strain, declining physical and mental health, and burnout (Baumbusch et al. 2022; Canadian Institute for Health Information (CIHI), 2018; Robinson et al. 2014). Responsibilities often include both activities of daily living (ADLs) and instrumental activities of daily living (IADLs), as well as managing behavioural and emotional changes associated with the disease (Alzheimer’s Association 2024; CIHI 2018). Despite a growing number of men in caregiving roles—particularly spousal caregivers—they remain underrepresented in dementia research, and their experiences, shaped by gender norms and masculine ideals, remain insufficiently understood (Donnell and Ryan 2011; Kluczyńska 2015).

Several studies have examined the gendered dimensions of caregiving, highlighting both the unique burdens men may experience and how they reconstruct identity through care (Knutsen and Råholm 2009; Sanders and Power 2009). A systematic review by Bueno and Chase (2023) found that while women experience higher psychological strain, men face greater difficulty adjusting to caregiving—particularly early in the disease trajectory. K. Lee et al. (2021) identified key contributors to caregiver burden in men, including IADL responsibility, concerns about health, and changes in the spousal relationship. Other studies, including Robinson et al. (2014) and Sanders and Power (2009), note that men often approach caregiving with a problem-solving mindset while simultaneously navigating shifts in marital roles and intimacy. Boyle (2014) found that men frequently struggle with domestic tasks like cooking—either avoiding them or assuming control in ways that limit their partners’ remaining agency. Hellström et al. (2017) describe how men internalize the caregiver role over time, evolving from reluctant participants to engaged caregivers. Yet this transition is often marked by emotional strain. Further, Brown et al. (2007) observed that older husbands are frequently reluctant to seek support, preferring to “shoulder” responsibilities alone.

Masculinity shapes men’s experiences of caregiving. In a study by Baker et al. (2010), they note that men often draw on workplace or managerial identities, emphasizing control and task completion—an approach that can limit emotional expression and help-seeking. Sandberg (2018) argues that even person-centred models may inadvertently reinforce normative gender roles through “re-gendering” practices. Other studies have shown that many men find meaning and growth in caregiving. Rykkje and Tranvåg (2019) describe a dynamic mix of loss and fulfillment, while Tolhurst and Weicht (2023) highlight how cultural ideals around independence, productivity, and restraint shape—and sometimes complicate—men’s caregiving practices.

Even though studies show that women typically experience greater caregiving burden (Xiong et al. 2020), men face distinct challenges, especially in reconciling traditional masculine expectations with the emotional demands of care (K. Lee et al. 2021). While men caregivers often report lower levels of stress, burden, or depression than women (Donnell and Ryan 2011; Knutsen and Råholm 2009), these differences may obscure hidden emotional strain, role conflict, and isolation. Studies suggest that masculine norms both constrain and motivate men’s caregiving approaches (Kluczyńska 2015; Sanders and Power 2009). Given these complexities, this paper explores the experiences of a man as he navigates caregiving for his wife with progressive dementia while residing together in the community.

### Theoretical Framework: Masculinity, Care, and Emotional Subjectivity

Gender and masculinity frameworks are instrumental in generating a more nuanced portrayal of men’s caregiving experiences through emphasizing “the diversity and differences within and across men’s lives” (Robinson et al. 2014, 423). These frameworks can help explain the variability in caregiving experiences and outcomes among men caregivers, while also connecting this research to broader concerns in men’s health, relational wellbeing, and identity formation. In this study, we draw on theoretical insights from Hanlon’s (2012) model of caring masculinities and Elliott’s (2016) and Hunter et al.’s (2017) conceptualizations of emerging masculine forms, to understand how men navigate caregiving in the context of dementia.

Hanlon (2009, 2012) regards caregiving as a multifaceted relational practice that interweaves physical, emotional, and mental work with identity, shaped by sociocultural norms. He identifies three masculine archetypes—*conventionalist, sharer, and carer*—to illustrate how men reconcile caregiving responsibilities with traditional masculine roles. *Conventionalists* adhere to traditional gender roles and breadwinning ideals; *sharers* aim to balance earning with caregiving; while *carers* assume primary care roles out of necessity, often experiencing profound shifts in identity through their care work. Hanlon contends that caregiving challenges hegemonic ideals but also reproduces them in complex ways, depending on men’s positionality, life stage, and relationship dynamics.

Building on this, Elliott (2016) developed a practice-based framework of caring masculinities, which foregrounds the rejection of domination and the integration of care values—such as emotional expression, interdependence, and relationality—into masculine identities. Caring masculinities resist hegemonic norms without abandoning masculinity itself. Rather than conceptualizing caring and hegemonic masculinities as binary opposites, Elliott argues for a more fluid and evolving model, where men can adopt caregiving roles and values without forfeiting their gender identity.

Hunter et al. (2017) expand on conceptualizing emerging masculine forms by examining the experiences of primary caregiving fathers, arguing that what is often framed as a shift toward caring masculinities may, in practice, reflect a broadening of hegemonic masculinity rather than its abandonment. They describe how caregiving men frequently negotiate a complex interplay between traditional provider roles and new caregiving expectations, maintaining links to masculine norms such as control, competence, and emotional restraint even as they adopt caregiving responsibilities. This suggests that caring masculinities may operate both as resistance to, and reformulation of, hegemonic ideals.

In the context of men caring for wives with progressive dementia, the theoretical perspectives described here allow us to explore how gendered expectations shape men’s experiences of role transition, emotional expression, and relational care. Dementia care often requires sustained emotional labour and renegotiation of intimacy, identity, and reciprocity—elements that challenge conventional masculine scripts. By further incorporating the sociology of emotions (e.g., Hanlon 2012; Held 2006; Tronto 1993), we also consider how emotional subjectivities are constructed within care relationships, and how these experiences shape a man’s understanding of being a caregiver. Emotions such as grief, love, frustration, and guilt are not only responses to caregiving but are also constitutive of masculine caregiving identities in transition.

By using these frameworks, we situate a husband’s caregiving experience for his wife with dementia within broader gendered structures and norms, while acknowledging the diverse, contingent, and relational ways in which men embody, resist, and rework masculinity through care.

## Methods

### Study Design and Setting

We performed a descriptive single case study drawing on data from a multi-year, longitudinal qualitative research project investigating the experiences of family caregivers of people living with dementia in community settings as they navigated caregiving roles and formal care systems. Case studies facilitate in-depth exploration of phenomena within real-life contexts (Baxter and Jack 2008; Crowe et al. 2011; Yin 2009), and are widely used in clinical practice and research to understand health and care-related issues—such as caregiving (Crowe et al. 2011; Heale and Twycross 2018). Longitudinal case studies are particularly effective in exploring experiences related to a chronic, progressive disease like dementia (Priya 2021; Thorsen et al. 2020).

This single case study enables close engagement with one man’s personal caregiving experience and how his masculine identities were negotiated over time; while the findings are not intended to represent men across all caregiving contexts or relationships, they offer in-depth insight into one situated narrative. The study took place in British Columbia, Canada, which has among the highest projected dementia growth rates in the country (Alzheimer Society of Canada 2024), and was conducted during the COVID-19 pandemic—a period of time that intensified stressors for people living with dementia and their caregivers (Hughes et al. 2021; Quinn et al. 2022).

Ethics approval for the broader study was granted by the University of British Columbia Research Ethics Board [H20-01093] and all procedures adhered to national guidelines for ethical research with human participants. Key ethical considerations included informed consent, confidentiality, secure data storage, and ensuring the emotional well-being of the participant, particularly given the sensitive nature of caregiving and dementia. This included offering breaks during the interview, reminding the participant that he could skip questions or stop at any time, and following up afterward to check in on his well-being.

### Recruitment and Sampling

Participants were recruited through word-of-mouth referrals, community newsletters, and social media advertisements. Eligibility criteria included: (a) being a primary caregiver for a community-dwelling person living with dementia, (b) residing in British Columbia, and (c) fluency in English. A single case was purposively selected to examine how one husband caregiver navigated his role, contributing insight into the gendered dimensions of care.

We investigated the experiences of a husband, Francis (pseudonym), caring for his wife, Irene (pseudonym), who was living with dementia. Francis was one of only two men in the larger sample and his sustained participation and co-residence with his wife enabled a rich, longitudinal account of home-based caregiving. The paucity of men participants in the larger study aligns with the literature indicating an underrepresentation of men in caregiving research (Poisson et al. 2023; Robinson et al. 2014). The other husband participant lived in an assisted living facility, whereas Francis’s case vividly illustrates the challenges of caring for a wife in a community setting with minimal supports. Francis’s regular diary submissions and interviews provided ample data on the caregiving experience.

### Case Participant

Francis, a 63-year-old retired man, is the primary caregiver for his wife, Irene, a 62-year-old woman diagnosed with early onset dementia in 2017. Francis has a college diploma, while Irene holds an undergraduate degree. They both come from a European family background and live together in their own home in a small, geographically isolated community of fewer than 30,000 people in British Columbia. Their annual family income ranges between US$41,000 and US$60,000.

While this study primarily focuses on gendered caregiving, we acknowledge that Francis’ and Irene’s experiences are shaped by intersecting factors, including age, early-onset dementia, socioeconomic status, geographic location, and cultural background. These influences, while not central to the analysis, were considered during interpretation to provide a more contextualized understanding of the case.

### Data Collection

Data were collected between November 2020 and Feburary 2023. Over this time period, six semi-structured interviews were conducted, and Francis submitted 17 narrative diary entries. Interviews were recorded and transcribed verbatim, ranging from 54 to 75 minutes in length (average: 68.5 minutes). Diaries were written in an open format in response to prompts and included reflections on caregiving experiences, health, service interactions, and highs and lows of daily life.

### Data Analysis

Data were analyzed using reflexive thematic analysis, initially informed by Hanlon’s masculinities and care framework (Braun and Clarke 2006, 2019; Hanlon 2009, 2012). In later analytic phases, we also drew on Elliott’s (2016) and Hunter et al.’s (2017) conceptualizations of caring masculinities to further analyze how caregiving intersects with shifting masculine identities. While our thematic analysis identified patterned meanings across the dataset, we also attended to the temporal and developmental dimensions of caregiving over time. This enabled analysis of how masculinity was negotiated in evolving circumstances, drawing conceptually from narrative thinking even as the method remained thematic. Our theoretical framing was also broadly situated within sociological theories of emotionality and identity. In particular, insights from the sociology of emotions (e.g., Held 2006; Tronto 1993) helped contextualize how emotional expression and relational dynamics are implicated in caregiving masculinities, even though these were not directly applied in the analytic process.

The six phases of thematic analysis included: (i) familiarization with the data, (ii) creation of initial codes, (iii) generation of preliminary themes, (iv) refinement of potential themes, (v) theme name and description, and (vi) report formulation. The first and second authors immersed themselves in the interview recordings and reviewed all transcripts and diary entries. Open coding was conducted in NVivo 12, a qualitative data analysis software, generating initial codes close to the data and relevant to the research question. These were then reviewed, grouped, and developed into themes, which were refined to ensure coherence with the dataset.

Throughout the analysis process, the authors engaged in collaborative reflection through discussions and memo-writing, helping to identify patterns and deepen interpretation. The analysis was conducted iteratively, with ongoing review of data, codes, themes, memos, and drafts of findings.

### Study Rigour

We ensured study rigour by establishing trustworthiness, a concept used in qualitative research to assess the adequacy of data collection, interpretation, and presentation (Ahmed 2024). Trustworthiness was achieved through credibility, transferability, dependability, and confirmability (Lincoln and Guba 1985). Credibility—confidence in the plausibility of findings—was supported by prolonged engagement, team debriefing, and the use of multiple data sources. Transferability— the degree to which the findings can be applied to other contexts— was enhanced through detailed descriptions of the study context and sample. Dependability—the repeatability of the findings within a similar participant group— was ensured by documenting procedures and maintaining an audit trail. Confirmability— confidence that the results could be validated by other researchers— was reinforced through triangulation and reflexive practices. Reflexivity was supported through ongoing dialogue and critical self-awareness, helping to surface potential biases and clarify the study’s purpose (Barrett et al. 2020; Guillemin and Gillam 2004).

## Findings

Over several years, Francis’s caregiving role evolves as Irene’s condition progresses. He provides a longitudinal perspective on how caregiving demands intensify and transform with time. The following themes illustrate the cumulative practical and emotional impacts of Francis’s extended journey: (1) The Practical Realities of Caregiving and (2) The Emotional Landscape of Caregiving. The first theme focuses on the functional, everyday challenges Francis experiences as he negotiates masculinity through overlapping roles of husband and caregiver in response to his wife’s progressing dementia. He takes on household duties, social planning, and caregiving tasks, balancing multiple responsibilities while searching for brief moments of respite in familiar routines. As Irene’s care needs grow, Francis continuously adapts, managing the practical realities of caregiving within a context shaped by prior shared domestic roles and shifting gendered expectations. The second theme explores the profound emotional shift accompanying Francis’s transition to ‘carer.’ The loss of familiar routines, emotional isolation, and communication struggles heighten his feelings of helplessness and frustration. Despite these challenges, Francis’s enduring expressions of commitment and love for Irene appear to anchor him, as he draws strength and hope from their bond amidst the complexities of caregiving. While the themes are presented as analytically distinct for clarity, the lived experience of caregiving is inherently multifaceted, therefore there is some thematic overlap, which is both expected and meaningful.

### Theme 1. The Practical Realities of Caregiving

Francis’s caregiving journey reveals the practical aspects of transitioning from the role of a ‘sharer’ – balancing caregiving with his responsibilities as a breadwinner and household partner –to that of a ‘carer,’ where caregiving becomes his primary focus (Hanlon 2009). This transition is not binary but iterative and relational, requiring Francis to continuously recalibrate his practices in response to Irene’s changing needs. As Irene’s dementia advances over time, Francis must take on new responsibilities, redefine his priorities to ensure her well-being, and maintain some semblance of routine in their shared life.

Throughout their journey, Francis looks for brief reprieves from his increasing responsiblities through familiar routines. By continuing his security duties at the local hockey rink and attending hockey games when he can, Francis maintains a link to his regular schedule while also receiving a brief break from caregiving demands. These activities, predominantly dominated by men, provide a source of stability and comfort for him:

I still provide security at the local hockey rink for our [local junior hockey team]. I look so forward to getting out a couple of hours a week to at least to attend the hockey games. I find that for that short time I get to forget my own troubles and get to enjoy life as we once knew it. (Diary entry– March 2022)

As Francis’s roles and responsibilities evolve, he assumes tasks that were once primarily Irene’s domain, balancing his identity as a husband with his expanding responsibilities as a caregiver. Friends and family offer practical assistance and emotional support, helping him navigate the new demands. Visits from Irene’s friends provide him with valuable respite time:

Irene’s friend flew in from Ottawa and spent two weeks with us. I got to spend time doing what I want to do, visiting with some of my buddies, going to hockey games, just things that restrict me usually. I'm very grateful that Irene has something out of the ordinary in her life as well (Diary entry– February 2022)

As Irene’s dementia progresses, Francis’s role continues to evolve to include increasingly complex caregiving tasks. Over time, he finds himself dedicating more hours to household duties and social planning. Each phase of Irene’s condition introduces new practical challenges that Francis must address, from helping to manage meals and household maintenance to coordinating visits and providing companionship. Although some of these responsibilities were previously shared, the shift toward near-full responsibility reflects a re-negotiation of existing roles rather than a wholly new division of labour. Francis reflects on how he has come to bear the full weight of duties they once shared:

I find that I'm now spending more time preparing meals and doing the routine things that we both did at one time, now more and more it becomes my sole responsibility. I often feel the weight has been placed on my shoulders and I have to carry everything myself. (Diary entry– December 2021)

When Irene’s memory and cognitive abilities decline, Francis steps in to manage most aspects of social planning and maintaining relationships that Irene can no longer sustain on her own. Visits from friends, once coordinated by Irene, now require his involvement to ensure they go smoothly. This marks a further transition from his role as a ‘sharer’ to a primary ‘carer,’ where he must anticipate and address her needs differently:

This past month we had Irene’s best friend and her husband visit us for two weeks. Since neither has seen her for almost a year, they both noted that she has declined in her memory loss. It was evident that she was having trouble with her short-term recall… she relied on me to do some of the things that she always did – like making meals, planning this day’s activities…(Diary entry– September 2021)

Other tasks that Irene previously managed independently, such as household duties, driving long distances, and handling their motorhome, now fall largely on Francis, advancing the transition from shared responsibilities to full caregiving. This shift not only adds to his practical workload but also heightens his sense of responsibility as he recognizes the full scope of his caregiving role and the demands it entails.

And I find that I end up doing now, you might say…I’d say 75 percent of the cleaning and cooking. And it gets tiring because I don’t know how my mom did it with us little savages when we were growing up… like, me, I’ve got to write a list out and say okay, I’m going to have this, this day, we’ll probably have leftovers so the next day we’ll make this, you know. And I’m always trying to be one step ahead. It doesn’t always work that way. (Interview– Feburary 2022)

With Irene’s condition impacting her memory and self-sufficiency, Francis’s narrative reveals an attempt at both continuity and adaptation in masculine identity—preserving a sense of competence and control while engaging in traditionally feminized care tasks. His transition from sharing responsibilities to assuming nearly all caregiving tasks illustrates the practical adjustments required in this shift, as he finds himself dedicating more time and energy to maintain consistency and security in their life together. Francis’s experience illustrates a tension between hegemonic masculinity—often associated with control, responsibility, and emotional restraint—and caring masculinity, which embraces empathy and emotional expression. Rather than fully adopting one model over the other, Francis continually shifts between these ways of being, depending on Irene’s changing needs and his own emotional responses. This shows that his caregiving role is not static, but an ongoing and dynamic negotiation of identity.

In later diary entries, Francis’s reflections expose a deeper struggle to cope with Irene’s declining memory and self-sufficiency. The journey’s longevity amplifies his feelings of helplessness, as he repeatedly encounters moments where he cannot help her regain what she has lost. His commitment to “live for that day” becomes a recurring theme, underscoring his resilience despite the overwhelming challenges he faces daily. This emotional labour is not simply a response to crisis, but a central mechanism through which Francis’s caregiving identity forms. This endurance, shaped and strengthened over time, becomes a key component of his emotional journey:

I find that lately I have to motivate her and worse check on little tasks that I had never had to do before. I so try not to be sad or sharp when everything around me is changing. I will never fully accept that “normal” as we once knew it does not exist. I think stress has aged me as I now feel older and less optimistic. Life is so full of surprises. We still have tomorrow, and we have to live for that day. (Diary entry– January 2022)

Here, Francis captures the weight of his transition from partner to primary caregiver, where the day-to-day realities of caregiving add up, testing his resilience. Yet, his commitment to “live for that day” reflects his pragmatic approach to embracing each new responsibility, meeting each day’s demands with acceptance and strength. Viewed through the lens of caring masculinities, Francis’s relational commitment and emotional expression coexist with, rather than diminish, his masculine identity. This orientation provides a sense of grounding, helping him navigate the loss of their “normal” by focusing on making each day meaningful.

### Theme 2. The Emotional Landscape of Caregiving

Francis’s caregiving journey is marked by a shift in the emotional landscape of his relationship with Irene over time. As he finds his way through the transition from sharer to carer, he must also navigate an increasingly challenging emotional environment. The once-familiar rhythms of their life together are transformed as Francis copes with the challenges of Irene’s dementia. Everyday activities, once simple and enjoyable, are now laden with difficulty, forcing Francis to balance his own needs with caregiving demands. The loss of familiar pastimes, such as attending hockey games, are considerable to Francis:

So, I mean she wants to be around me all the time. I used to go to the hockey games. There’s no hockey games. So, I would give her a bit of a break, I had a break. No, there’s no games anymore and if there is, you can’t go. So, I mean, you’re just, it seems like everything you’ve had is now just a memory. Everything we’ve done and known in life, it is gone. And you have to figure out how you’re going to go through this in today’s society. (Interview– November 2020)

As Francis assumes responsibilities formerly held by his wife—such as trip planning and decision-making—he describes the pressure to maintain composure and resilience while having to confront waves of overwhelming emotions, leading him to question the validity of his own emotions in his new role:

I think it makes me anxious. A lot of times it makes me tired or even depressed. And it’s like, why am I depressed? There’s nothing I should be depressed about. And then I have to get back out of that frame of mind to go, oh, there’s no reason for feeling that way. Just smarten up. You’ve got to be in charge here. You’ve got to be the one that’s the caregiver, not the other way around anymore, you know? (Interview– Feburary 2022)

Francis’s emotional vulnerability is exposed as he contrasts his current struggle with Irene’s illness against the resilience he once demonstrated in the face of extreme situations in his past work. Now, he finds himself feeling helpless and heartbroken:

I see it’s getting bad and I go to bed and I read till she falls asleep and then I end up crying myself to sleep. I’ve witnessed prison hostages, murders, riots, that didn’t bother me. This bothers me. I can’t do anything to fix it. I can’t do anything to make it better for her. (Interview– May 2021)

This shift signals a reconfiguration of emotional norms, where expressions of grief and vulnerability—typically restricted under hegemonic masculinity—become integral to his caregiving identity.

Watching Irene’s cognitive decline, Francis becomes overwhelmed by a sense of loss and powerlessness. His diary entries reveal poignant memories of time spent with Irene’s frail father, highlighting the emotional weight of witnessing her deteriorating health:

Overall, I saw a big change in Irene’s memory and independence. It was a social and beautiful visit with her aging father who is frail and diagnosed with Alzheimer’s as well. I felt down the road, I will be dealing with much more depressing and anxious times. The frustration and total helplessness were sometimes overwhelming. Irene has good days and bad days. I feel her pain but feel so helpless. (Diary entry–August 2021)

Communication struggles further intensify Francis’s emotional burden when Irene’s short-term memory loss leads to misunderstandings, frustration, and moments of regret:

Like, the other day I apologized to her. I snapped. I said, “Jesus Christ, [Irene]. I don’t know what I can do short of tattooing it on your friggin arm, you know?” And then she starts crying. She says, “If I can’t talk to you, who can I talk to? We’re not going to communicate; we’re not going to be able to be a couple if you don’t work with me on this. Do you think I like this? Do you think this is fun for me? Do you think this is easy for me? I know it’s difficult for you but you’ve got to work with me in this. I didn’t ask for this.” And then you just feel like shit and you end up crying with her, you know what I mean? (Interview– November 2020)

As both Francis and Irene grapple with their own fears and challenges. Irene’s plea for understanding and partnership calls Francis to a place of empathy, reflecting the depth of their bond and the emotional strength required to navigate the challenges ahead. He recalls Irene’s unwavering support during his most difficult times, which stirs feelings of obligation and gratitude:

Yeah. I… I mean I had some…I’ve been diagnosed with post-traumatic stress disorder twice in my life through the pen (penitentiary or prison). Both times Irene was there for me. … She was there for me, every waking moment of 30 years of hell. If I can’t be with her for the few years that I have left, I’m not worthy of anything. [pause] Sorry, it’s an emotional thing for me. I find that I’m a lot more emotional lately than I have been in my life. (Interview– Feburary 2022)

This reciprocity highlights the interdependent emotional fabric of care, where love and duty merge. Francis’ feelings of frustration, guilt, grief, and love are not peripheral but central to his evolving identity as a man caregiver.

With the continued progression of Irene’s dementia, Francis finds himself facing the emotional challenges of caregiving alone. Initially, he struggles to understand and process these feelings, but as time passes, he comes to realize the depth of emotional isolation his role entails. Reflecting on how their relationship has transformed, he expresses a sense of loneliness as he assumes a supportive role without the reciprocal comfort Irene once provided:

My partner always was there to talk to and ease my frustrations. Now I feel that I can’t burden her with my feelings as she has so much on her plate. The saddest part is that Irene is aware of the situation, and she knows that in time it will only get worse. (Diary entry– August 2021)

The emotional complexities of being labeled as a “caregiver” also weigh heavily on Francis. He expresses resistance to the term, perceiving it as diminishing the depth and equality of their relationship:

To me, it’s demeaning. It’s demeaning for the reason that I feel like I’m babysitting. And that’s not the case. It’s, I would say, how is it to cope with somebody who’s not [pause] the same as they were before? I…just the word “caregiver” is to me taking care of somebody who’s like a child. And maybe down the road, that’s going to be the thing. But right now, I don’t want to face that, I don’t want to recognize that, and I hate being a caregiver. (Interview– Feburary 2022)

Francis’s discomfort with the caregiver label reflects a broader tension experienced across caregiving roles and a gendered dynamic in which caregiving is constructed as incompatible with masculinity. His resistance signals not only a disruption of established relational identities, but also a rejection of the symbolic feminization of his role, even as he enacts caregiving in practice.

Amid these emotional hardships, Francis’s enduring commitment to Irene, grounded in their 30-year partnership, offers a glimmer of hope. His reflection on the strength of their relationship affirms his resolve to remain by her side despite the inevitable challenges ahead:

…we have no choice. You’ve got to have some hope. You’ve got to have something. Like I already told Irene, “I don’t even want to imagine life without you. I mean you’re my best friend for thirty years. Honey, you’re…you know. (Interview– May 2021)

Francis’s journey from ‘sharer’ to ‘carer’ reveals a deep emotional trajectory. His experience encompasses a range of emotions—from despair, helplessness, and frustration, to empathy and resilience. Through his candid reflections, Francis brings to light the vulnerable, multifaceted nature of caregiving, where feelings of nostalgia, loss, and a deep commitment to love intermingle, creating an emotional landscape marked by love, heartbreak, and hope. His account illustrates that masculinity is not abandoned but reshaped through care, highlighting the relevance of caring masculinities and the socially constructed nature of emotion in reshaping gendered caregiving identities. These emotional responses are deeply contextual—rooted not only in gendered expectations, but also in the evolving demands of caregiving and the enduring bond of a long-standing partnership.

## Discussion

Francis’s journey reflects a shift from Hanlon’s (2009) *sharer* to *carer* archetype within the caring masculinities framework. Initially, Francis balances additional household chores and caregiving tasks with personal interests like attending hockey games and spending time with his friends, embodying the *sharer* archetype. As Irene’s dementia progresses, Francis transitions into the *carer* identity—taking on the relational, emotional, and physical labor central to Hanlon’s framework. This shift is not only practical but reflective of a deeper transformation in masculine identity, where caregiving influences self-reconstruction and emotional subjectivity. The transition is neither immediate nor complete; rather, Francis’s experience reflects an ongoing negotiation of roles, shaped by relational change and gendered expectations over time.

Although caregiving by men is often described as task-oriented (Donnell and Ryan, 2013; Robinson et al. 2014), Francis’s narrative suggests a more complex negotiation between traditional masculine roles and emotional engagement. In line with Rykkje and Tranvåg’s (2019) findings, Francis adapts to and broadens his daily responsibilities even when faced with tasks that fall outside conventional masculine norms. He takes on additional housework, financial duties, and personal care responsibilities, while reflecting on how the changes in their dynamic influences him as a man and as Irene’s husband, much like Hellström et al.'s study (2017), where the men gradually internalized their caregiving roles over time. These shifts further support Elliott’s (2016) notion of caring masculinities, where men incorporate care and relationality into masculine identity without necessarily abandoning masculine self-understandings.

Francis’s difficulties with managing finances and transportation—tasks traditionally associated with men—complicate hegemonic assumptions. Rather than reinforcing masculine competence, Francis’ challenges reveal how caregiving disrupts fixed gender scripts and forces a redefinition of roles. Hanlon (2012) emphasizes that such disruptions are not merely personal but socioculturally mediated—care practices are shaped by intersecting structures such as class, gender, and age, which constitute men’s social location (Tolhurst and Weicht 2023) and influence how they navigate or reproduce hegemonic masculinity. As Kluczyńska (2015) notes, men who reconcile caregiving with masculine self-perceptions often do so by redefining care itself as a masculine practice, allowing for pride and self-esteem in the carer role. Francis’s experience reflects this tension, shaped by his age, relational history, and the gendered expectations that contour his caregiving journey.

As Irene becomes less able to offer emotional support, Francis experiences deepening isolation. He relies cautiously on friends and family, reflecting research showing men often have smaller, less-utilized social networks (Pöysti et al. 2012). This reliance—while offering respite—also underscores how caregiving reconfigures emotional landscapes. Sandberg (2018) reminds us that normative gendered expressions can become de-stabilized in dementia contexts. Francis’s emotional vulnerability is central to his reconfiguring of masculinity and caregiving. His experience disputes assumptions that men carers are less emotionally affected (K. Lee et al. 2021) and aligns with the broader sociological view that emotions are not only personal, but also socially constructed and identity-forming (Hanlon 2012; Tronto 1993).

Francis’ disclosure of emotions such as fear, grief, and frustration, contrast sharply with the stoic detachment often associated with hegemonic masculinity. Yet, as Hunter et al. (2017) argue, caring masculinities may not entirely resist hegemonic norms but can expand or reconfigure them. Francis maintains a strong sense of control and responsibility, but now this is expressed through his care and commitment, not detachment. His account reflects both resistance to and reworking of dominant masculine ideals, suggesting fluidity rather than rupture.

Francis’ caregiving experience is embedded within a relational history of couplehood. As Hellström et al. (2017) and Rykkje and Tranvåg (2019) emphasize, *couplehood*—the shared sense of being a couple—persists even as the partner’s cognitive abilities decline. Francis’ commitment is grounded not only in duty but in love, intimacy, and shared life history. His narrative affirms the importance of understanding caregiving as an extension of relational identity and interdependence, particularly in long-term spousal relationships.

Francis’s resistance to the “caregiver” label reflects the complex and sometimes stigmatizing ways caregiving is culturally framed. For him, the term may evoke connotations of infantilizing or one-directional care—more akin to “babysitting”—rather than acknowledging the mutual, relational dimensions of spousal support. As seen in Tolhurst and Weicht’s (2023) study, this discomfort is further shaped by dominant associations of caregiving with femininity, which can marginalize men’s experiences. Together, these tensions point to the need for a broader cultural reframing of caregiving—one that recognizes it as a gender-inclusive and emotionally meaningful practice, rather than a role implicitly tied to women or reduced to task-based service.

In summary, Francis’ experience invites a relational and contextual understanding of men who are caregivers of a spouse with dementia, attuned to theoretical insights on masculinity, emotion, and identity. His journey challenges the narrow framing of men as strong, emotionally-distant caregivers and reinforces the value of integrating gendered qualitative research to capture the nuanced ways men embody, resist, and reshape caregiving roles.

### Implications for Policy and Practice

We suggest three key policy and practice implications. (1) Establish support networks tailored to men caregivers, addressing their unique challenges such as emotional isolation and reduced social networks (J. Lee et al. 2021; Pinquart and Sörensen 2006). Community-based programs and support groups for men can foster shared experiences and provide emotional support, dementia education, coping strategies, and stress management. (2) Offer flexible respite care options to help men caregivers balance caregiving duties with personal interests, preventing burnout and promoting well-being (Bueno 2024; Pöysti et al. 2012). Accessible respite services enable caregivers to take necessary breaks and help to preserve their personal identities. (3) Promote public awareness campaigns and healthcare training programs that challenge traditional gender norms, supporting an inclusive view of masculinity and normalizing emotional vulnerability in caregiving (Donnell and Ryan 2013). Policymakers should invest in educational programs for healthcare professionals and stakeholders to recognize and support the diverse emotional experiences of men caregivers (K. Lee et al. 2021).

### Limitations and Directions for Future Research

The findings presented here are drawn from a longitudinal, single-case study of a husband caregiver, limiting their transferability of findings to a broader population of men caregivers. The use of a single case was intentional, allowing for a deep, contextualized understanding of how one man navigates care within a specific relational and cultural context. However, this focus on a partner-caregiver whose spouse remains at home limited our ability to illustrate broader caregiving patterns or represent the full diversity of masculinities in care. Future research should include larger, more diverse samples of men caregivers, such as adult sons, to better understand their distinct experiences, challenges, and support needs, particularly the impact of parental bonds on caregiving (Bueno 2024; Donnell and Ryan 2013; McDonnell and Ryan 2014). As our study focused on a community-dwelling participant in British Columbia, Canada, it may not fully capture the experiences of men caregivers in other living arrangements (e.g., nursing homes, assisted living) or regions with differing healthcare systems and cultural norms. Further research should examine how cultural expectations shape caregiving roles for men across diverse backgrounds to inform tailored interventions (Donnell and Ryan 2013; Pöysti et al. 2012; Robinson et al. 2014). Additionally, future studies should explore caregiving experiences across a spectrum of gender identities and family roles, considering intersections with socio-demographic factors such as race and class (K. Lee et al. 2021; Xiong et al. 2020).

## Conclusion

This study highlights the emotional and practical complexities faced by a husband caring for his wife with dementia. Francis’s story illustrates that caregiving is not a simple role shift but an ongoing negotiation of identity, shaped by emotional strain, evolving responsibilities, and long-term relational bonds. Rather than remaining emotionally detached, Francis expresses feelings of grief, frustration, and fear. His reluctance to identify as a “caregiver” and his limited engagement with support networks reveal the tension between lived experience and cultural narratives about gender and care. These dynamics demonstrate how caregiving can challenge and reshape how men understand themselves in relation to masculinity and emotional expression. Francis’s experience exemplifies how men may take on unfamiliar caregiving roles while navigating traditional expectations of self-reliance and strength. While his story is unique, it underscores the broader need for a more inclusive understanding of men’s caregiving experiences and more responsive forms of support and recognition.
